# Case Report: A Bilateral Synchronous Primary Non‐Small Cell Lung Cancer Patient With Two Different EGFR Mutations

**DOI:** 10.1002/cnr2.70319

**Published:** 2025-08-25

**Authors:** Thanh‐Dung Quach, Kien Hung Do, Dinh Tung Nguyen, Hyeon‐Gyu Yi, Thi‐Phuong Vu, Tran Thanh Tin, Nghi Le Vinh, Son Hai Vu

**Affiliations:** ^1^ Oncology Center Vinmec Times City International Hospital, Vinmec Healthcare System Hanoi Vietnam; ^2^ Internal Medicine Residency Program, College of Health Science VinUniversity Hanoi Vietnam; ^3^ Department of Medical Oncology 1 Vietnam National Cancer Hospital Hanoi Vietnam; ^4^ Department of Pathology Vinmec Times City International Hospital, Vinmec Healthcare System Hanoi Vietnam; ^5^ Vinmec‐VinUni Institute of Immunology, College of Health Sciences VinUniversity Hanoi Vietnam

**Keywords:** afatinib, EGFR mutation, non‐small cell lung cancer

## Abstract

**Background:**

Non‐small cell lung cancer (NSCLC) represents the majority of lung cancer cases, with epidermal growth factor receptor (EGFR) mutations playing a crucial role in disease prognosis and treatment. Around half of Asian patients with NSCLC, particularly non‐smoking women, have EGFR mutations. These patients with NSCLC typically exhibit a single EGFR mutation in exon 18, 19, 20, or 21. It is extremely rare for patients with bilateral primary NSCLC to harbor two different EGFR mutations.

**Case:**

We report a case of 70‐year‐old non‐smoking Vietnamese female patient diagnosed with synchronous bilateral primary NSCLC, each harboring different EGFR mutations—G719C in exon 18 in the right lung and an exon 19 deletion in the left lung. The patient underwent surgical resection of the left lung lesion, followed by targeted therapy with afatinib for the right lung lesion, which resulted in tumor reduction and disease stability for 1 year before disease progression.

**Conclusion:**

Our study underscores the complexity of diagnosing and managing synchronous bilateral NSCLC with distinct genetic profiles. This report also highlights the importance of comprehensive molecular profiling to select an optimal treatment strategy to improve patient outcomes.

## 1 Background

Lung cancer is the most common malignancy disease with the highest incidence of mortality worldwide [1]. Non‐small cell lung cancer (NSCLC) accounts for more than 80% of all lung cancer cases, in which *EGFR* mutations are commonly found [1, 2, 3, 4]. The prevalence of *EGFR* mutations differs significantly regarding race and geographical location, with incidences of 10%–15% reported in European and North American countries and 26% in Latin America [5]. Multiple clinical trials have revealed the presence of activating mutations of EGFR in about 50% of Asian patients with NSCLC [6, 7]. Rare mutations include point mutations, deletions, and insertions in exons 18–21 accounting for approximately 5% of the cases [8, 9]. Afatinib is the 2nd generation EGFR‐TKIs that covalently bind to EGFR and irreversibly block signaling from ErbB family receptors [10, 11]. Concrete clinical results from LUX‐Lung 2, LUX‐Lung 3, and LUX‐Lung 6 have promptly led to FDA approval of afatinib as a first‐line treatment for patients with EGFR mutation and other uncommon mutations [12, 13]. In lung cancer, multiple mutations in EGFR are rare, including bilateral synchronous NSCLC characterized by two primary lung tumors. These primary tumors may share similar or different pathological characteristics [14, 15, 16]. It has been sporadically documented that several *EGFR*‐mutant NSCLC patients may carry concomitant genetic aberrations in different oncogenic driver genes such as *EGFR* and *ALK* [17, 18].

## 2 Case Report

**FIGURE 1:**
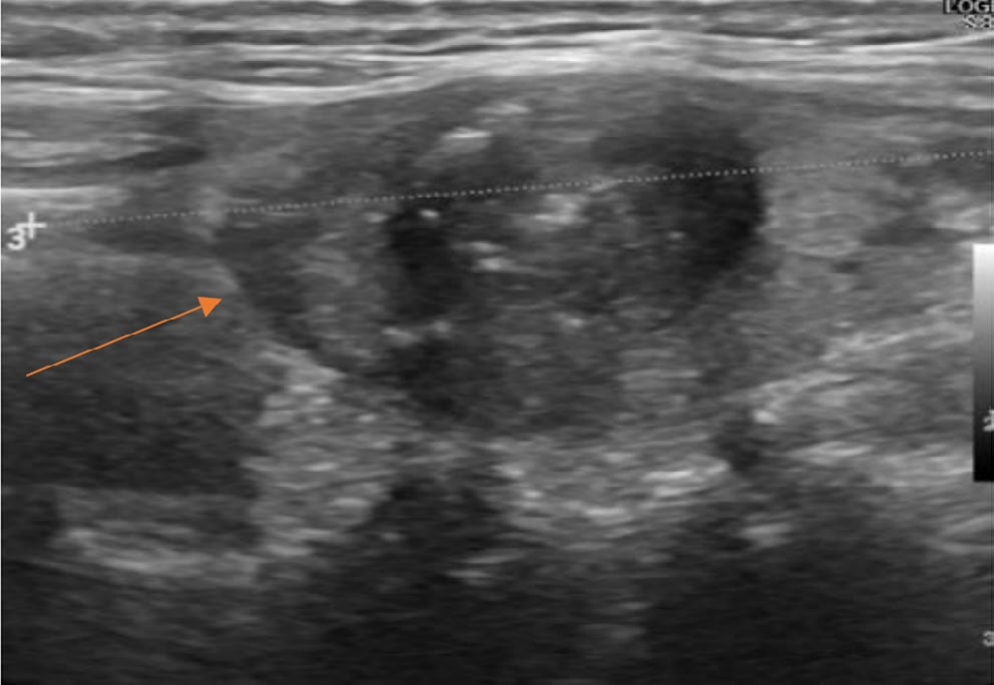
Thyroid ultrasound. A thyroid nodule measuring 14 × 20 × 20 mm (ACR‐TIRADS 5), is identified. The lesion is highly suspicious for malignancy, with potential capsular invasion.

**FIGURE 2:**
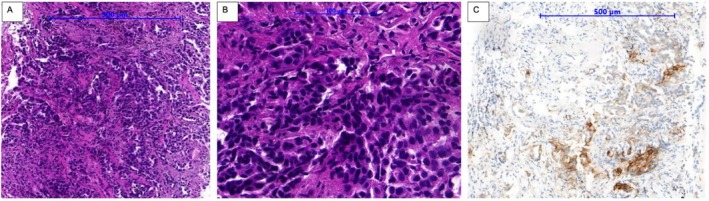
Histopathology of right lung core biopsy. HE staining of the biopsy sample of the right lung mass, (A) (×50), (B) (×200) demonstrate adenocarcinoma with a predominant solid growth pattern characterized by sheets of neoplastic cells and the absence of other distinguishable adenocarcinoma subtypes including lepidic, acinar, papillary, or micropapillary architectures; (C) Immunostaininghistochemistry (PD‐L1 ×50) using SP‐263 antibody shows approximately 30% PD‐L1 positive viable tumor cells exhibiting the partial or complete brown‐staining membrane, relative to all viable tumor cells present in the sample.

In August 2023, a 70‐year‐old previously healthy non‐smoking female presented to Vinmec Times City International Hospital, Hanoi, Vietnam for a health checkup due to a 5 kg weight loss over the past 5 months, accompanied by intermittent dry cough. She had no complaints of shortness of breath or chest pain. On clinical examination, the patient was alert, oriented, and had an ECOG performance status of 1. There were no abnormal findings on physical examination, including peripheral lymph nodes, thyroid, and lung auscultation. When the patient underwent a thyroid ultrasound, this revealed a hypoechoic nodule in the left thyroid lobe measuring 14 × 20 × 20 mm, classified as ACR‐TIRADS 5 (American College of Radiology Thyroid Imaging Reporting and Data System). Fine‐needle aspiration (FNA) of the lesion was performed, leading to a diagnosis of papillary thyroid cancer, staged as cT1N0M0 (c‐stage I). The patient was advised to undergo either a thyroidectomy or radiofrequency ablation, but she then refused treatment and opted for routine surveillance (Figure 1). At the same time, a computed tomography (CT) scan of the chest revealed bilateral lung masses. A transthoracic biopsy of the right lung mass was performed, and histopathological analysis confirmed adenocarcinoma with a predominant lepidic growth pattern (Figure 2).

**FIGURE 3:**
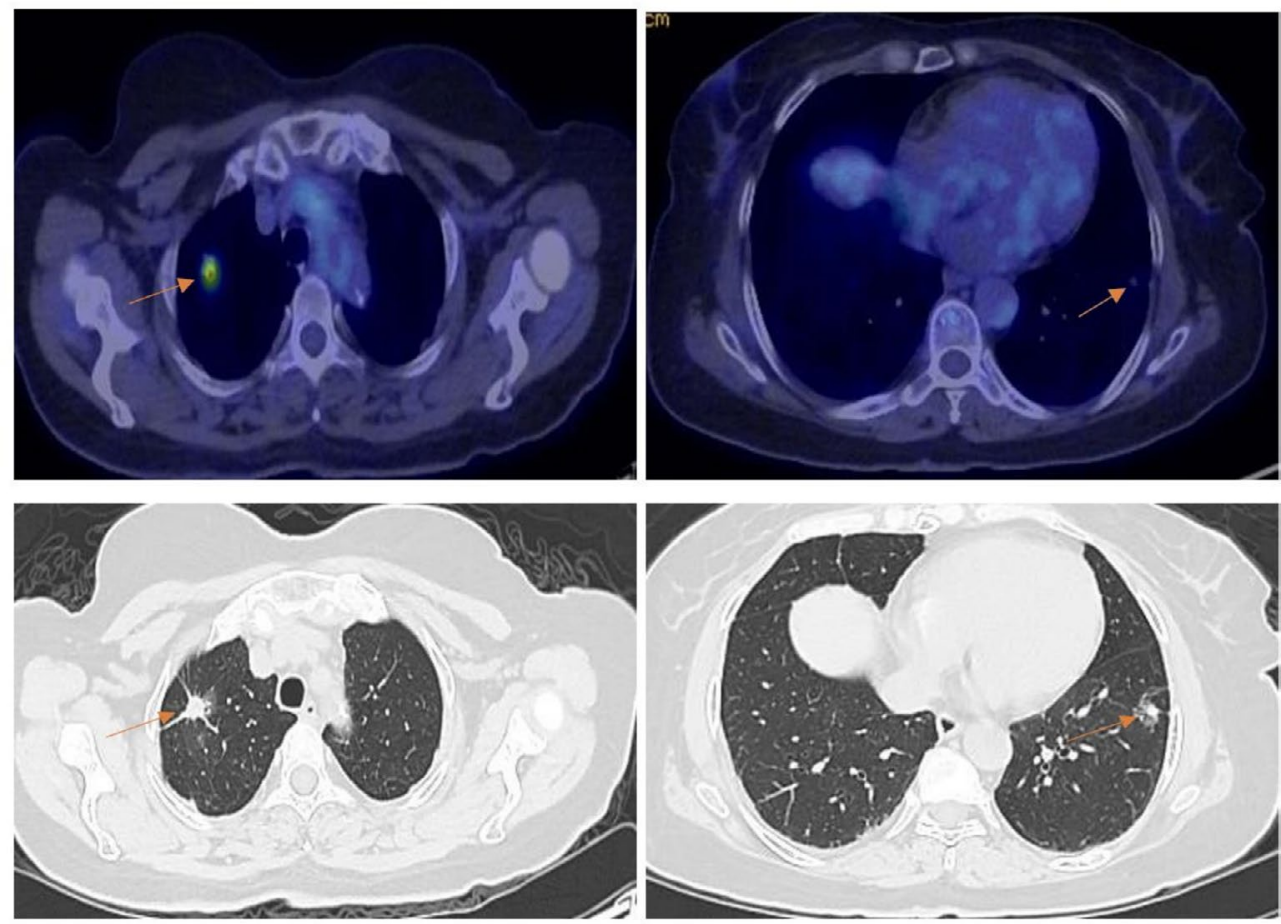
PET‐CT of bilateral primary lung masses. PET‐CT revealed a spiculated solid nodule in the right upper lobe with increased FDG uptake (SUVmax: 7.3), measuring 15 mm in diameter, and a part‐solid nodule with irregular borders was detected in the left lower lobe, measuring 15.3 mm with mildly increased FDG uptake (SUVmax: 1.3).

Further investigation using PET‐CT scan revealed an increased Fluorodeoxyglucose (FDG) uptake (SUV max: 7.3) spiculated solid nodule with the largest diameter of 15 mm at the upper lobe of the right lung (Figure 3) Additionally, an irregular bordered part‐solid nodule with the largest diameter of 15.3 mm was identified in the lower lobe of the left lung, with slightly increased FDG metabolism (SUVmax: 1.3) suggesting second primary lung cancer. Several lymph nodes with increased FDG metabolism (SUVmax: 4.5), with a large node measuring 10 × 12 mm, were found in the right hilum. An irregular hypodense lesion with increased FDG uptake (SUVmax: 11.3) was observed in the left thyroid lobe (data not shown). Chest CT scan also showed bronchiectasis with interstitial thickening and ground‐glass opacities at the base of the right lung, consistent with a chronic inflammatory lesion.

The substitution mutations of G719C in exon 18 were detected in the right lung tumor using EGFR XL StripAssay. Based on the imaging characteristics of the left lung lesion, it was necessary to differentiate it from other diseases such as metastatic lung cancer, pulmonary tuberculosis, inflammatory pseudo‐tumor, and lymphoma. Unfortunately, biopsy examination could not be performed due to the limited tumor size and restricted location.

The patient was then diagnosed with non‐small cell right lung cancer cT1bN1M0 (stage IIB), adenocarcinoma with lepidic component, EGFR (+) exon 18 (G719C belongs G719Xsubstitutions)/non‐small left lung cancer cT1bN0M0 (stage IA2)/Papillary Thyroid cancer cT1N0M0. After thorough discussion at the tumor board of our hospital, a right lobectomy with lymph node dissection and a left lung resection with frozen biopsy were the consensus recommendation. However, the patient and her family opted for minimally invasive surgery on the left side only due to concerns about surgical complications, especially post‐operative pain. The patient underwent video‐assisted thoracoscopic wedge resection of the left lower lobe lesion. Intraoperative findings revealed a 1 cm solid nodule infiltrating the visceral pleura. Frozen section confirmed adenocarcinoma. Station 10 hilar lymph node dissection was performed.

**FIGURE 4:**
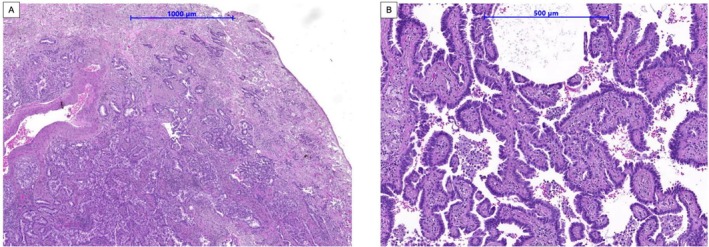
Histopathology of the left lung surgical specimen. HE staining of (A) (×20) the oval to round glands that invade the fibrous stroma accounting for around 20% of the entire tumor sample is the acinar pattern of adenocarcinoma, and (B) (×50) of the papillary pattern of adenocarcinoma consists of tumor cells arranged around the fibrovascular cores that make up approximately 80% of the entire tumor sample.

Postoperative pathology confirmed adenocarcinoma in the left lung with papillary component predominance and PD‐L1 positivity, with a tumor proportion score (TPS) of 30% (Figure 4). Additionally, EGFR mutation exon 19 deletion was detected using EGFR XL StripAssay.

**FIGURE 5:**
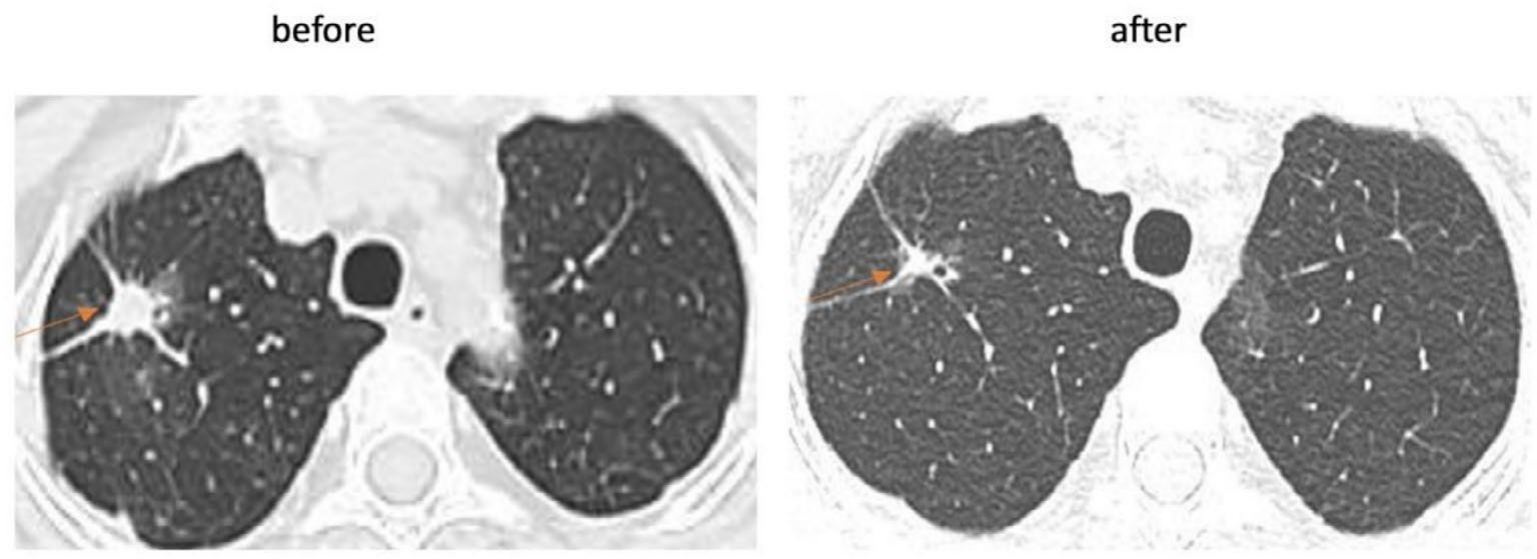
CT scan after 3 months of afatinib therapy. CT scan shows significant size reduction of the right upper lobe tumor after 3 months.

**FIGURE 6:**
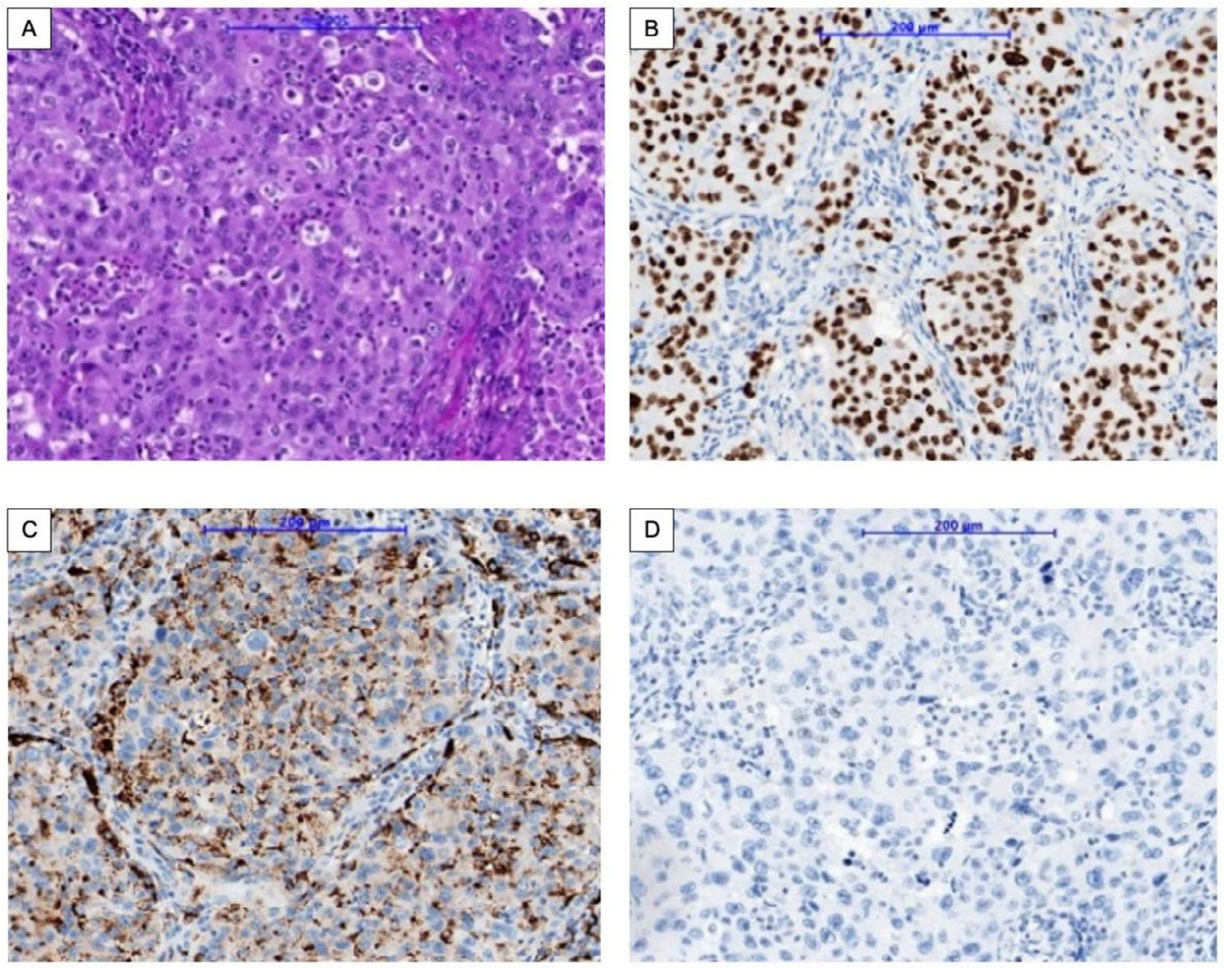
Histopathology of the right lung surgical specimen. H&E staining (A, ×100) shows a solid growth pattern composed of polygonal tumor cells arranged in cohesive sheets, closely resembling the morphology observed in the prior biopsy specimen (Figure ). Immunohistochemistry demonstrates diffuse TTF‐1 (B, ×100) and Napsin A (C, ×100) expression, with absence of p40 staining (D, ×100), supporting a diagnosis of solid‐pattern adenocarcinoma and excluding squamous cell carcinoma.

**FIGURE 7:**
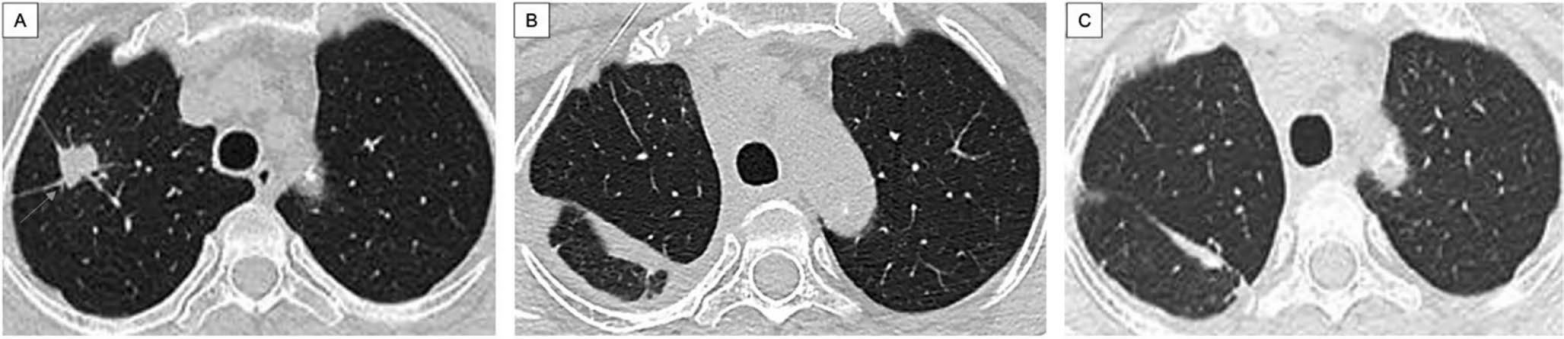
CT scan of the right lung at recurrence and during post‐treatment follow‐up. (A) Enlargement of the right upper lobe tumor, indicating disease progression. (B) One month after right upper lobectomy, no residual or secondary pulmonary lesion was observed; a small right pleural effusion was present. (C) One month after completing four cycles of adjuvant chemotherapy, no new lesions were detected.

After recovery from the first surgery, the patient was fully informed about the curative treatment options and the most appropriate therapeutic approach for her condition. Despite thorough consultation with the medical oncologist in charge, she declined surgical intervention. Additionally, she opted against radiation therapy after being adequately informed of the associated risk of radiation‐induced pneumonitis. Ultimately, she chose to pursue systemic therapy with oral TKIs based on the provided recommendation. According to NCCN guidelines for NSCLC, osimertinib is often recommended to patients with common EGFR mutations including exons 19 deletion mutation, or exon 21 L858R mutation. However, our patient has an uncommon EGFR mutation in exon 19 (G719C mutation that belongs to G719X). Therefore, afatinib was recommended and administered to the patient, positively resulting in tumor reduction after 3 months (Figure 5). Stable disease was achieved for 1 year; then the right lung tumor started to progress while no other lesions were observed. A second surgery was again recommended. Learning from the first attempt, we incorporated psychological counseling into the preoperative planning for the second surgery, and the patient consented to the planned surgery. She underwent right upper lobectomy with lymph node dissection. Postoperative pathology revealed a poorly differentiated carcinoma with lymphovascular invasion, but no lymph node involvement (pT2N0M0) (Figure 6). Given the high risk of recurrence, the patient received four cycles of adjuvant chemotherapy with pemetrexed and carboplatin. Follow‐up CT imaging after 3 months showed stable disease (Figure 7). As of May 2025, the patient remains clinically stable and reports no new symptoms. Key clinical events are summarized in Table 1.

**TABLE 1:** Timeline of clinical events.

Time	Event
08/2023	Diagnosed with papillary thyroid carcinoma (cT1N0M0); incidental bilateral lung nodules detected on chest CT. Right lung biopsy confirmed adenocarcinoma.
30/08/2023	Underwent wedge resection of the left lower lobe.
09/2023	Cnfirmed synchronous primary lung adenocarcinomas (EGFR G719C and exon 19 deletion) and thyroid cancer.
10/2023–9/2024	Initiated afatinib 30 mg/day; achieved partial response with stable disease maintained for 12 months.
10/2024	Radiologic progression of right upper lobe tumor following 12 cycles of afatinib; surgery indicated.
11/2024	Underwent right upper lobectomy with lymph node dissection.
12/2024–5/2025	Received 4 cycles of adjuvant chemotherapy with pemetrexed and carboplatin.

## 3 Discussion

This case report presents a rare incidence of synchronous bilateral lung adenocarcinomas with EGFR driver mutations at exons 18 and 19. The presence of discordant mutations supports the diagnosis of two independent primary tumors and highlights the complexity of lung cancer pathology and management challenges.

Multiple primary lung cancers (MPLCs) are classified into synchronous MPLCs, where multiple primary tumors appear simultaneously, and metachronous MPLCs, where tumors develop at different times. Most MPLC cases are diagnosed at early stages, making surgical intervention a common treatment option. However, for patients with impaired pulmonary function or multiple nodules, novel systemic therapies including targeted therapy and immunotherapy are crucial [19, 20].

To contextualize our finding, we conducted a literature review using Scopus with the following search queries: TITLE‐ABS‐KEY‐AUTH (“synchronous bilateral lung cancer” OR “bilateral lung cancer” OR “synchronous lung tumors”) AND (LIMIT‐TO (LANGUAGE, “English”)). This search returned 49 results, from which we selected 25 articles relevant to our case: an adult with different types of lung cancers. Among these articles, only a few cases describe synchronous MPLC with different EGFR mutations. The novelty, malignancy status, treatment regimen, and outcome of summarized cases are highlighted in Table 2. Unlike previously reported discordant EGFR mutations, the coexistence of G719C and exon 19 deletion observed in our case has not been documented.

**TABLE 2:** Highlights of reported cases of synchronous NSCLC with discordant EGFR mutations.

No	Age/gender	Smoking status	Primary malignancy	Second primary malignancy	Clinical significance	References
1	70F	Never	Adenocarcinoma in right inferior lobe: EGFR G719C mutation in exon 18	Adenocarcinoma in left lower lobe: EGFR exon 19 deletion	First case reported of this combination. Left lung mass was treated with lobectomy. Treatment of right lung mass with afatinib resulted in a partial response, followed by stable disease for 1 year then disease progression. The patient was successfully treated with wedge resection of the left lower lobe.	This study
2	78M	Passive smoking	Non‐mucinous adenocarcinoma in right inferior lobe cT1aN0M0: EGFR exon 19 micro deletion (c.2240_2254del15)	Mucinous adenocarcinoma in the right upper lobe cT1bN0M0: no EGFR mutation, negativity for keratin 20 and CDX2, positivity for keratin 7	The patient refused surgery and was successfully treated with stereotactic body radiation therapy.	Linhas et al. [21]
3	43F	Ex‐smoker	Adenocarcinoma in right lower lobe: EGFR L858R mutation in exon 21	Squamous cell carcinoma in right lower lobe intermingled with the primary mass: EGFR L858R mutation in exon 21, novel EGFR S768I mutation in exon 20	Combination of histologic transformation with new EGFR S768I mutation. EGFR TKI resistance developed after 8 months of response with gefitinib.	Longo et al. [22]
4	69M	Never	Adenocarcinoma in right middle lobe: EGFR G719A mutation in exon 18	Adenocarcinoma in left upper lobe: EGFR L858R mutation in exon 21	Successfully treated with surgery.	Sakai et al. [23]
5	77F	Ex‐smoker	Adenocarcinoma in right upper lobe: EGFR G719A mutation in exon 18	Adenocarcinoma in left upper lobe: EGFR L858R mutation in exon 21	Successfully treated with surgery.	

Overall, this case presentation highlights the diagnostic and therapeutic complexity of synchronous multiple primary lung adenocarcinomas with discordant EGFR mutations. Comprehensive histopathologic and molecular testing, combined with psychological counseling, is essential to guide treatment strategies and contributes to favorable outcomes in patients with molecularly heterogeneous lung cancers.

## Author Contributions

Conceptualization: T.D.Q., S.H.V. Investigation: T.D.Q., D.T.N. Validation: K.H.D., H.G.Y. Writing – original draft: T.T.T., D.T.N., T.D.Q., S.H.V. Visualization: T.P.V., N.L.V. Writing – review and editing: T.D.Q., S.H.V. Project administration: T.D.Q. Formal analysis: D.T.N., T.T.T., T.P.V. Methodology: T.P.V., K.H.D., H.G.Y., T.D.Q.

## Disclosure

Submitting an article indicates that the content has not been previously published or is not currently being reviewed for publication elsewhere. All authors, as well as the relevant authorities at the institution where the research was conducted, have given their approval for publication.

## Consent

Informed consent from the patient has been obtained.

## Conflicts of Interest

The authors declare no conflicts of interest.

## Data Availability

The data that support the findings of this study are available from the corresponding author upon reasonable request.
